# Potential toxicity of polystyrene microplastic particles

**DOI:** 10.1038/s41598-020-64464-9

**Published:** 2020-04-30

**Authors:** Jangsun Hwang, Daheui Choi, Seora Han, Se Yong Jung, Jonghoon Choi, Jinkee Hong

**Affiliations:** 1 Department of Chemical and Biomolecular Engineering, Yonsei University, 50 Yonsei-ro, Seodaemun-gu, Seoul, 03722 Republic of Korea; 2 School of Integrative Engineering, Chung-Ang University, 84, Heukseok-ro, Dongjak-gu, Seoul, 06974 Republic of Korea; 3 Division of Pediatric Cardiology, Department of Pediatrics, Yonsei University College of Medicine, Seoul, 03722 Korea

**Keywords:** Cell culture, Environmental monitoring, Supramolecular polymers

## Abstract

Environmental pollution arising from plastic waste is a major global concern. Plastic macroparticles, microparticles, and nanoparticles have the potential to affect marine ecosystems and human health. It is generally accepted that microplastic particles are not harmful or at best minimal to human health. However direct contact with microplastic particles may have possible adverse effect in cellular level. Primary polystyrene (PS) particles were the focus of this study, and we investigated the potential impacts of these microplastics on human health at the cellular level. We determined that PS particles were potential immune stimulants that induced cytokine and chemokine production in a size-dependent and concentration-dependent manner.

## Introduction

Microplastic particles can be divided into two categories, primary and secondary. Plastic particles less than 5 mm in diameter are considered microplastics^1^. Although local and national governments in North America took action in 2015 to regulate the manufacture of microbeads, microplastic particles are still produced in other parts of the world^2^. Primary microplastic particles are intentionally manufactured at the microscale and are key ingredients in scrubs^3^, handwashing soaps^4^, cleansers^5^, toothpastes^6^, and biomedical products^7^. Primary microplastic particles, particularly those between 1 and 5 µm in diameter, are spherical and often made of polypropylene (PP), polystyrene (PS), or polyethylene (PE).

Unlike primary microplastic particles, secondary microplastic particles are generated through the fragmentation of plastic litter^8–10^. Plastic debris is the primary source of secondary microplastic particles found in the ocean and soil, because the debris breaks down into mesoparticles and macroparticles. Ultraviolet (UV) radiation from the sun and physical forces degrade these particles into plastic microparticles and nanoparticles^11,12^. A recent study investigated the fragmentation of PS coffee cup lids, disposable plates, and PS foams irradiated with simulated UV light to determine the degradation mechanism^13^.

Seafood is also a potential source of particulate plastic contaminants^14–18^. Anthropogenic debris, including plastic particles and fibers, was found in over 20% of individual shellfish and the gastrointestinal (GI) tracts of fish in a 2015 study^19^. The ingestion of microplastics by fish and shellfish has been demonstrated in several studies^16,18,20^.

Food, food containers, everyday products (personal care products), biomedical products, and drinking water are not the main sources of particulate plastic contaminants. However, they may be continuous sources of plastic particles^21–24^. For example, one study found microplastic fragments in all types of returnable and single-use plastic bottles^24^. Other examples include facial scrubs that are commonly used for exfoliation. It is estimated that 1.1 million women in the UK use these scrubs every day. A typical amount for daily use is 5 mL, which contains between 4,594 and 94,500 microplastic particles^4,5^. Additionally, three out of four body exfoliants contain microplastics. These primary plastic particles have the potential to pass into the sewage system^4^, and only 25% of them are filtered out of water sewage treatment plants^4,25^. Therefore, direct contact with microplastic particles in everyday products is a potentially serious problem. According to one study, PS particles from laboratories may be a source of primary plastic particulate contaminants^26^. In this study, we focused on PS nanoparticles and microparticles found in the surrounding environment. Depending on their size, shape, and functional group chemistry, ingested microplastic particles can cause various problems. Microplastic particles cannot be digested, so aggregates containing biomolecules and microplastics or nanoplastics can cause gastrointestinal dysmotility or obstruction. It is well known that size is an important cytotoxicity parameter *in-vitro*^27,28^. In a recent study, 30 nm COOH-PS particles in seawater aggregated in less than 30 minutes^28^. The hydrodynamic diameter of nanoplastic particles increases with an increase in the NaCl concentration. The hydrodynamic diameter of PS nanoparticles (NPs) is ~100 nm when the ionic strength of NaCl is low (1–50 mM). However, PS NPs have been found to aggregate when the NaCl concentration is higher^29^. PS NPs are thus expected to aggregate readily in seawater. Their interactions with various impurities can harm aquatic animals and may cause side effects in humans. Absorbed microplastics and nanoplastics less than 1.5 µm in diameter can damage cells directly. NPs were recently obtained *via* the degradation of PS microplastics after 56 days simply by irradiating them with UV light, which was three times faster than degradation without UV irradiation^13^. These findings suggest that simple chemical forces can generate nanoscale particles from PS particles and lead to direct cell damage. Several studies have reported that microplastics <1.5 µm in diameter can penetrate tissues and result in the accumulation of microplastics^6,30,31^. Anywhere from 1% to 4% of PS particles in the intestine are thought to migrate to the bloodstream. The translocation of nanoparticles is thought to be very low, and the most probable sites of accumulation are Peyer’s patches in small intestine^32^. However, it is possible that the transfer of nanoplastics into the bloodstream after ingestion could lead to local inflammation or induce allergic reactions in tissues^29,33–35^. The aggregation of microplastics and nanoplastics with biomolecules and chemicals often has toxic effects. According to the guidelines of the World Health Organization (WHO), human exposure to styrene monomers should be limited to a time-weighted average (TWA) of 20 ppm (85 mg/m^3^) with a short-term exposure limit (STEL) of 40 ppm (170 mg/m^3^)^36^. Chemicals used to synthesize PS particles, such as monofunctional peroxides, may also cause toxicity. Initiators like benzoyl peroxide and azobisisobutyronitrile are used to reduce polymerization time. Other chemicals used for PS synthesis include catalysts, such as zeolites and iron (III) oxides; emulsifiers; and stabilizers likebis(2,2,6,6-tetramethylpiperidin-4-yl) decanedioate. These chemicals are found throughout the world and are considered environmental contaminants. They accumulate in the food chain, predominantly in the fatty tissues of animals. The majority of microplastics and nanoplastics consumed by humans are found in food, food containers, and water^18,19,31^. Everyday products and contaminated soil can also be sources of primary microplastics, and their ingestion by humans may cause health problems^4,37^. The average person ingests approximately 11,000 microplastic and nanoplastic particles annually by consuming seafood, such as oysters, crabs, and fish^18,38^. Returnable and single-use plastic bottles may contain as many as 15 macroparticles or nanoparticles per liter^24^. Microparticles with sizes ranging from 1 to 500 µm have been found in drinking water. Fifty percent of microplastics and nanoplastics are below 1.5 µm in diameter. These particles are found in fibers, fragments, and spherical foams^24,39,40^, which indicates that spherical foam microplastics and nanoplastics could be primary plastic particles. It has also been suggested that PS microparticles account for less than 10% of the plastic particles in untreated water and sediment^39^. Therefore, monitoring primary PS particles could reveal the origins of these pollutants.

Polystyrene is a colorless, transparent polymer composed of styrene monomers and has a specific gravity of 1.04–1.07 g/cm^3^. PS is soluble in organic solvents, such as ketones, esters, and aromatic hydrocarbons. It is resistant to acids, alkalis, salts, mineral oils, organic acids, and alcohols^41^. As a hard and solid plastic, PS is often used to manufacture transparent products, such as food packaging and laboratory ware. Lightweight polystyrene foam provides excellent thermal insulation for many applications, such as roofing, building walls, refrigerators, and freezers.

In this study, we focused on the potential impact of primary PS particles on human health based on the size and concentration of the particles rather than the effects of individual chemicals. We evaluated the potential of primary PS particles to cause toxicity at the cellular level. Although many organizations and research groups have investigated the effects of primary PS microparticles and nanoparticles on marine ecosystems^42–44^, it is unclear what effects primary PS particles have on humans. Spherical primary PS particles are used for a wide range of biomedical applications that directly affect humans, such as drug delivery^45^, imaging^7,46^, and labware. Exploring the relationship between primary PS particles and potential risks to human health is thus important for understanding PS particle toxicity. In this study, we evaluated the potential of primary PS microparticles and nanoparticles to cause toxicity in humans based on size and concentration and investigated whether the PS particles mediated immune responses and allergic reactions.

## Results and discussion

**Figure 1:**
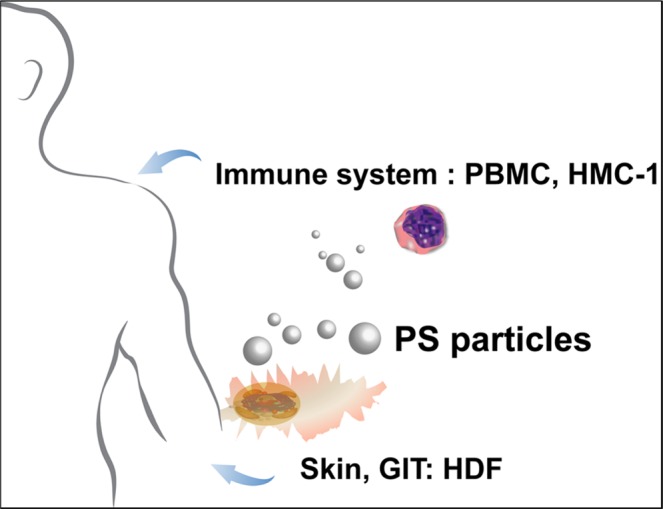
Illustration of the PS particle intake pathways of three cell lines. Human intake of PS particles from personal care products can occur *via* absorption through theskin. Intake can also occur through the ingestion of PS particles infood, food containers, drinking water, or biomedical products. We evaluated the potential of primary PS microparticles and nanoparticles tocause toxicity in humans based on the size and concentration of the particles in human cells.

We hypothesized that humans couldingest PS particles from everyday products, food, biomedical products, food containers, and drinking water^24,38^. We tested six different sizes of PS particles using Human Dermal Fibroblasts (HDFs), Human Peripheral Blood Mononuclear Cells (PBMCs), and the Human Mast Cell line (HMC-1)to determine their cytotoxic potential (Fig. 1).

### Characterization of PS particles

**Figure 2:**
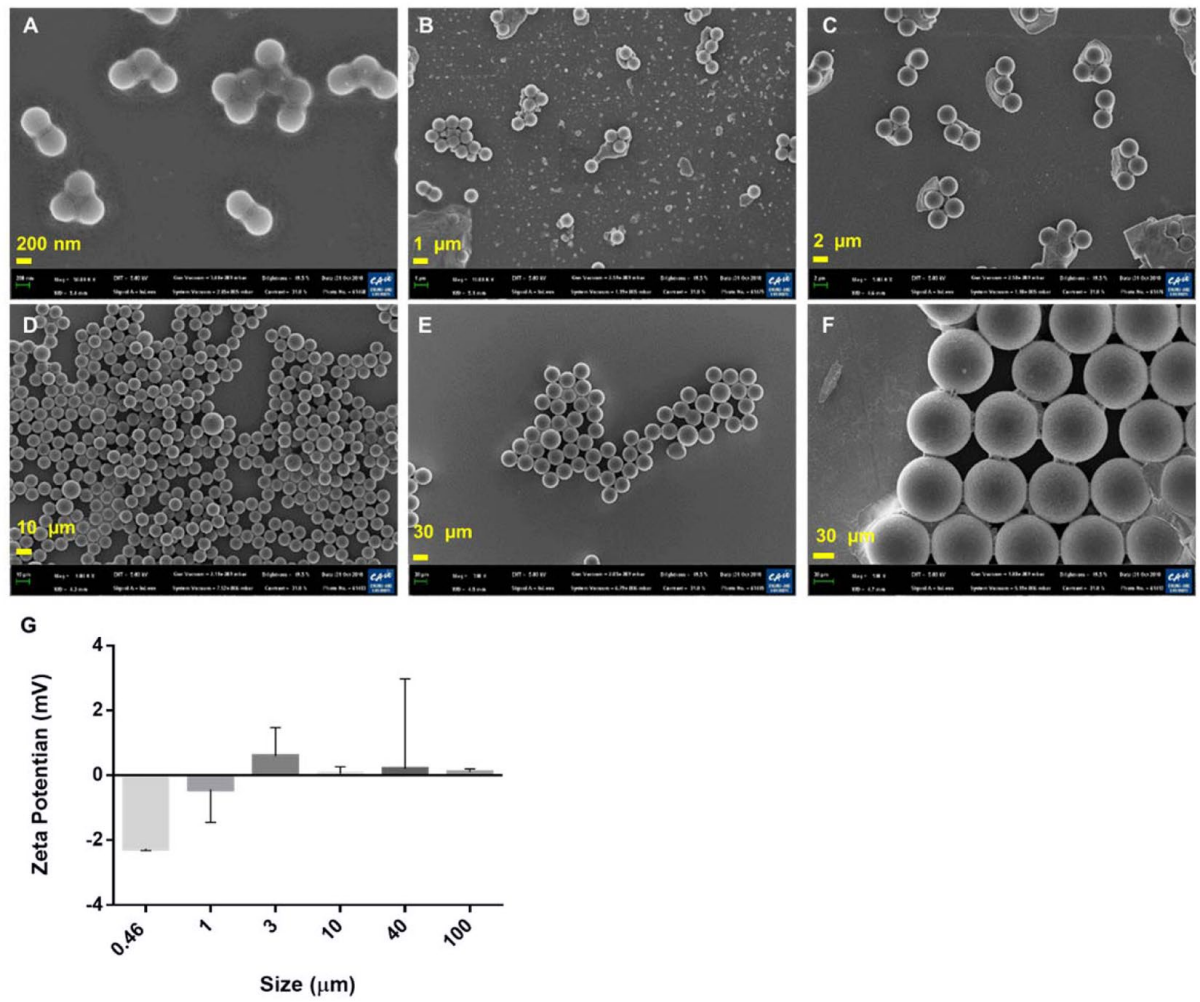
SEM images and zeta potentials of PS particles. (**A**) 460 nm PS nanoparticles. (**B**) 1 µm PS particles. (**C**) 3 µm PS particles. (**D**) 10 µm PS particles. (**E**) 40 µm PS particles. (**F**) 100 µm PS particles (scale bar = 200 nm, 1 µm, 2 µm, 10 µm, and 20 µm). (**G**) Zeta potentials of the PS particles.

Scanning electron microscope (SEM) images were used to examinethe morphologies of individual platinum-coated PS particles (Fig. 2A–F) in aggregates. The smaller particles were more likely to aggregate due to Van der Waals interactions with Na^+^or Ca^2+^ in the buffer. The zeta potential of 460 nm PS particles was −2.2 ± 0.1 mV (Fig. 2G–F), while the zeta potentials of the other PS particles were closer to zero. The formation of PS nanoparticle aggregates could be explained by the Derjaguin-Landau-Verwey-Overbeek (DLVO) theory. According to the DLVO theory, small particles carry less charge than large particles at pH 7. The electrical double layer (EDL) repulsion forces between small particles at a given ionic strength are thus smaller^47^. PS particles with small negative charges tended to move closer to one another as the ionic strength of NaCl increased to 137 mM in PBS buffer. In addition, all of the PS particles were uniform in size (Fig. 2D–F).

#### Cytotoxicity tests

**Figure 3:**
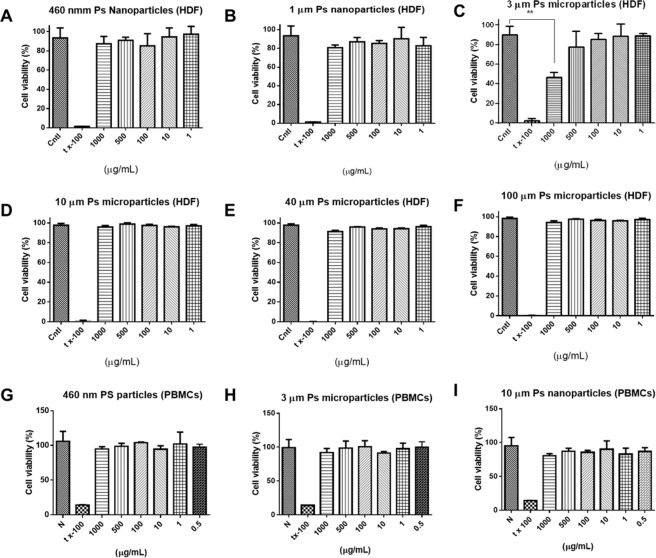
Cytotoxicity of PS particles. (**A**) 460 nm PS nanoparticles on HDFs. (**B**) 1 µm PS particles on HDFs. (**C**) 3 µm PS particles on HDFs. (**D**) 10 µm PS particles on HDFs. (**E**) 40 µm PS particles on HDFs. (**F**) 100 µm PS particles on HDFs. (**G**) 460 nm PS nanoparticles on PBMCs. **(H**) 3 µm PS particles on PBMCs. (**I**) 10 µm PS particles on PBMCs.

We investigated the responses of human-derived HDFs, HMC-1 cells, PBMCs, and other cells to the PS particles. HDFs are the predominant cells in stromal tissue, which plays an important role in wound healing process and also provides a protective barrier to prevent the absorption of PS particles. Human mast cells were selected for this study, because they exhibited many of the key characteristics of tissue mast cells. These included the expression of histamine, tryptase, and heparin, which could indicate a close relationship between PS microparticles, the human immune system, and hypersensitivity^48^. The behavior of isolated PBMCs, such as the expression of cytokines, could provide unique information about the human immune response to PS particles in the body. The cells were completely coated with PS particles (1 mg/mL) during treatment. A lack of HDFs toxicity might indicate that primary PS particles are less damaging to organs and skin. None of the PS particles caused significant cytotoxicity in the HDF cells or the PBMCs (Fig. 3) at concentrations up to 500 µg/mL. We also included a PS concentration above 500 µg/mL in the experimental design. The viability of HDF cells treated with 3 µm PS particles at a concentration of 1,000 µg/mL was reduced by 40% (**p < 0.001), while the viability of the PBMCs did not decrease. Cell viability profiles against PBMCs are shown in Fig. 3G–I. It could be concluded that PS particles are not cytotoxic to HDFs and PBMCs in usual condition but might cause damage to skin in extreme high concentration condition.

The intake of PS particles through food, daily products, and biomedical products was estimated. According to the Sigma datasheet, the average weight of a 3 µm PS particle was 1.5 × 10^−8^ mg. Based on reported data, we calculated amaximum intake of 11,000 plastic particles per person annually through food. Humans can potentially consume up to 325 plastic particles per liter of drinking water. Based on the recommendation to drink two liters of water per day, a person may consume up to 237,250 plastic particles per year. A maximum annual intake of 248,250 plastic particles including plastic particles from drinking water could thus be expected^18,39,49–51^, which could be converted to 4 µg/year assuming a PS particle size of 3 µm. We calculated the annual intake of PS particles assuming that the specific gravities, sizes, and shapes of the plastic particles varied. The maximum annual consumption per person can exceed 133 mg/year if the plastic particles are larger than 100 µm in diameter (More than plastic particles in size 100 µm represents a volumetric increase of 33^3^ compared to 3 µm particles whereaspecific gravity is 1.04–1.07 g/cm^3^). The maximum intake of PS particles from personal care or biomedical products based on a 5 mL volume of product ranged from 4,594 to 94,500 particles per day^4,52,53^. Based on this range, we estimated that up to 35 × 10^6^ primary plastic particles were being used annually. Assuming a particle size of <3 µm or >100 µm, this was equivalent to a primary plastic particle intake of 0.5–18,860 mg per person through scrubbing alone. We thus increased the estimated amount of total human exposure to primary plastic particles to 0 to19,000 mg y^−1^L^−1^. It has been reported that less than 10% of plastic waste is comprised of PS particles^39^. We calculated an average individual PS particle intake of 0–19 mg y^−1^ L^−1^, or 0–19 µg/mL, with particles ranging in size from nanometers to micrometers. We assumed the PS particles were applied over a given area at the maximum concentration for a specific time to monitor the biological response.

### Confocal imaging

**Figure 4:**
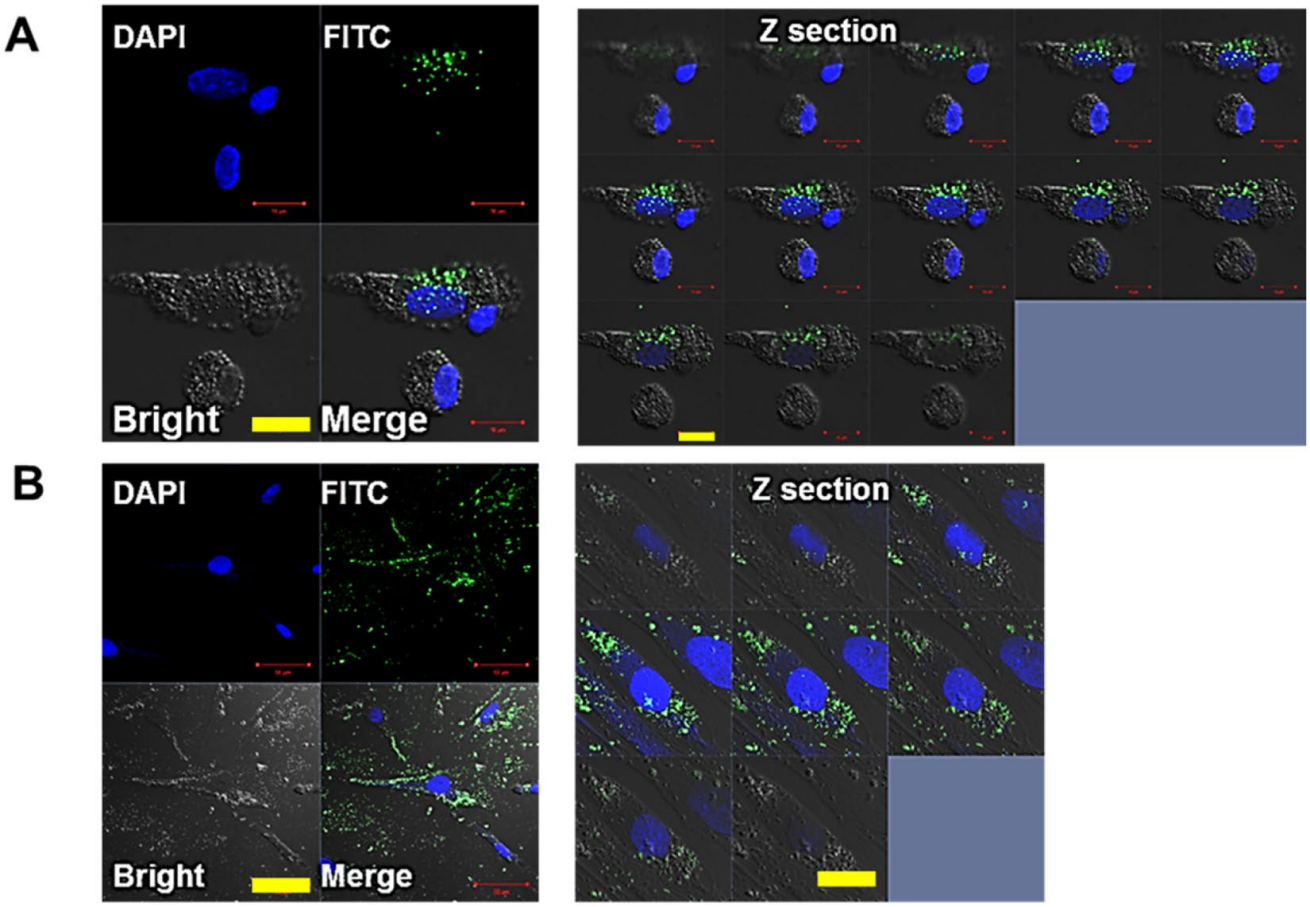
Confocal images of PS particles in cells. (**A**) Fluorescent images of 460 nm PS-FITC nanoparticles in PBMCs after DAPI staining (scale bar = 10 µm). Right: Z-section images. (**B**) Fluorescent images of 460 nm PS-FITC nanoparticles taken up by HDFs collected after DAPI staining (scale bar = 50 µm). Right: Z-section images.

The mechanism of cellular uptake depends on the size and surface charge of the particles. Uptake of particles smaller than 700 nm occurs *via* receptor-mediated endocytosis^54^, whereas larger particles are taken up *via* phagocytosis^55^. NH_2_-terminated polystyrene nanospheres have been reported to be highly toxic to RAW 264.7 macrophages, epithelial cells, and human microvascular endothelial hepatoma cells^56^. This was attributed to the deposition of particles in the cytosol, which caused an increase in mitochondrial Ca^2+^ uptake and cell death. Negatively charged polymeric nanoparticles less than 500 nm in diameter tended to accumulate efficiently in mice tumors^57^. Based on these results, 460 nm PS-FITC particles were chosen for our study. PS microparticles could be transformed into nanoparticles^13^, so we thought the results of these studies would be helpful for understanding the toxicity of PS particles. We also tested to determine whether 460 nm PS particles induced a different biological response. The 460 nm FITC-labeled PS nanoparticles allowed us to determine the location of particles within the cells after endocytosis (Fig. 4). The PS-FITC particles were mainly located in the cytoplasm of phagocytic cells, such as neutrophils and macrophages, whereas phagocytosis by lymphocyte-like cells was not indicated (Fig. 4A) in the Z-section images^57^. Similar to our observation in the PBMCs, the PS-FITC particles in HDF cells were mostly located in the cytoplasm, which indicated successful intake of the particles (Fig. 4B).

#### Hemolysis test

**Figure 5:**
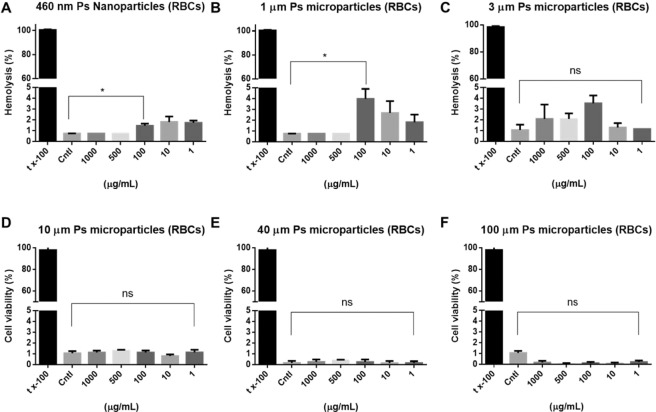
Hemolysis of RBCs after contact with PS particles. (**A**) 460 nm PS nanoparticles. (**B**) 1 µm PS particles. (**C**) 3 µm PS particles. (**D**) 10 µm PS particles. (**E**) 40 µm PS particles. (**F**) 100 µm PS particles. 5% tx-100 served as the positive control. Cntl indicates no treatment. Absorbance was measured at 540 nm.

An *in-vivo* hemolysis assay was performed to evaluate the compatibility of PS particles with blood, which would enable us to identify severe acute toxic reactions in the RBCs^58^. Hemoglobin is an iron-containing oxygen-tranport metalloprotein, which has a significant role in the transport of oxygen from lungs to cells and tissues^59^. Good correlations between *in*-*vitro* hemolysis assays and *in*-*vivo* toxicity have been demonstrated in several studies^60,61^. The results of these studies suggest that polymers are generally harmful to cells, although the magnitude of toxicity depends on the concentration, exposure time, and cationic nature of the polymers. Microplasticparticles less than 5 µm in diameter exerted hemolytic effects on RBCs owing to their surface charges and aggregation in highly saline buffer (*p < 0.03, Fig. 5A–C). Aggregates of plastic particles and biomolecules release chemicals that also have cytotoxic effects^62–65^. We investigated RBC hemolysis after direct contact with PS particles in different concentrations and sizes. PS particles more than 10 µm in diameter cannot penetrate blood vessels. However, the observed hemolytic effects indicated that direct contact resulted in cytotoxicity. PS particlesless than 5 µm in diameter had a hemolytic effect of approximately 4% relative to the control. This implied that the smaller particles had a stronger tendency to aggregate due to size and high concentration had effect on RBC hemolysis. The induction of hemolysis depended only on size, not concentration. PS particles smaller than the RBCs, which had an average diameter of 6–8 µm, were more cytotoxic at each concentration due to their large surface areas. In contrast, large PS particles did not have any hemolytic effect on RBCs (Fig. 5D–F). Although the hemolysis index shown in this study was not evident^66^, hemolysis was related with particle size in negative correlation. Thus, the hemolytic adverse effect *in vivo* of nanoparticles should be further investigated, especially for the small nanoparticles.

#### Cytokine profiles

The risks of microplastic ingestion in animal tests depend on the degree of exposure and the size of the affected area^16^. Translocation, redistribution, and retention are major concerns^67,68^. As with pollen and dust, direct contact with plastic particles may induce primary body defense mechanisms for ejection, such as tearing, sputum production, sneezing, and coughing^69^. Microparticles in the small intestine resulting from absorption through the skin and by cells were transferred to other bodily tissues *via* blood vessels in animal tests, wherecell-mediated defense mechanisms occurred^67^. In such cases, plastic microparticles can be detected in the lumen of blood and lymph vessels within minutes^70,71^. Absorption of smaller plastic nanoparticles and microparticles in the digestive tract proceeds *via* pinocytosis and vesicular phagocytosis by phagocytes^72–74^. These processes depend on particle size. The findings of several studies suggest that plastic microspheres 50–100 nm in diameter are more readily absorbed through Peyer’s patches and villi in the gut than particles with larger diameters of 300–3,000 nm^75^, and that surface charge and hydrophilicity increase the uptake affinity^76–78^.

In this study, we evaluated the cytokine release profiles of immune cells to determine whether inflammation could be triggered by treatment with PS particles. We also investigated whether cytokine release occurred in a size- or concentration-dependent manner. Interleukin 2 (IL-2) is one of the most common cytokines, and it is involved in the control of cell tolerance and immunity. IL-2 is a T-cell growth factor (TCGF) that has been detected insupernatants obtained from mitogen-stimulated peripheral blood lymphocytes. IL-2 is produced predominantly by activated CD 4^+^ an CD 8^+^T lymphocytes^79^. TNF-α serves as an immune mediator for cell adhesion, migration, angiogenesis, and apoptosis. TNF-α is a pro-inflammatory cytokine produced by bone marrow-derived cells, such as primarily by macrophages and also by broad types of cells (lymphocytes, mast cells, endothelial cells and so on), after stimulation with various agents^80^. The upregulation of these cytokines is a potential indicator of an immune response and inflammation. IL-6 acts as both a pro-inflammatory cytokine and an anti-inflammatory myokine. IL-6 is produced in response to infections and tissue injuries, and it contributes to the host defense by stimulating the acute-phase response^81^. IL-10 is an anti-inflammatory cytokine that inhibits the activity of Th1 cells, NK cells, and macrophages during infection^82^.

**Figure 6:**
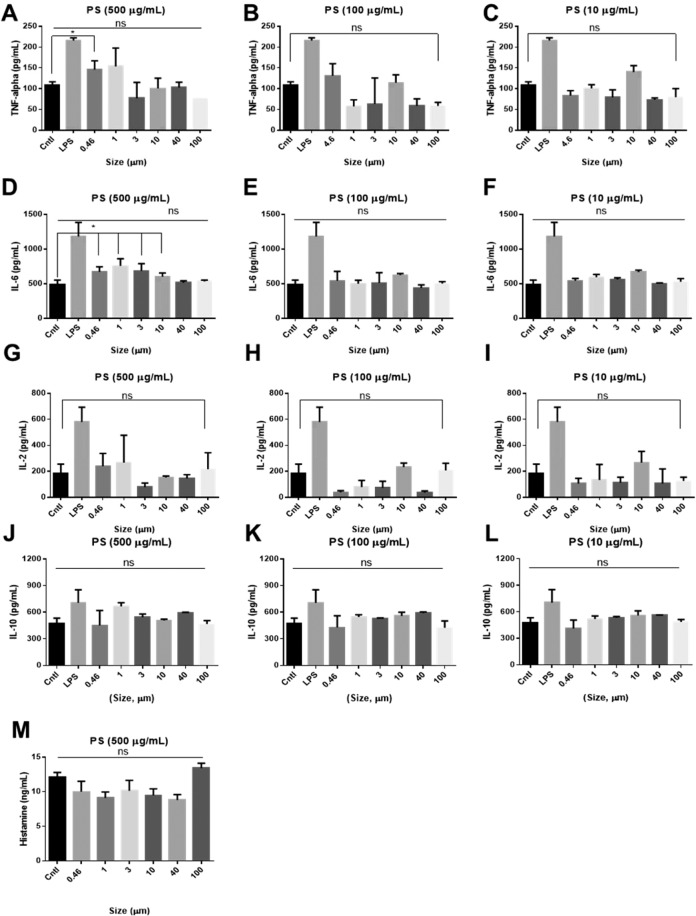
Cytokine profiles of TNF alpha, IL-2, IL-6, IL-10, and histamine. TNF-α secretion induced by PS particles of various sizes at concentrations of (**A**) 500 µg/mL, (**B**) 100 µg/mL, and (**C**) 10 µg/mL. IL-2 secretion induced by PS particles of various sizes at concentrations of (**D**) 500 µg/mL, (**E**) 100 µg/mL, and (**F**) 10 µg/mL. IL-6 secretion induced by PS particles of various sizes at concentrations of (**G**) 500 µg/mL, (**H**) 100 µg/mL, and (**I**) 10 µg/mL. IL-10 secretion induced by PS particles of various sizes at concentrations of (**J**) 500 µg/mL, (**K**) 100 µg/mL, and (**L**) 10 µg/mL. (**M**) Histamine profiles after treatment with 500 µg/mL PS particles of different sizes. Cntl: no treatment. LPS: 2.5 µg/mL.

The ELISA results (Fig. 6A–C) showed an increase in the secretion of TNF-α by treatment with PS particles less than 1 µm in diameter at a concentration of 500 µg/mL (*P < 0.03) and a significant change in the secretion of IL-6 by treatment with PS particles less than 10 µm in diameter at a concentration of 500 µg/mL (*P < 0.04, Fig. 6D–F). However, IL-2 secretion by treated cells and the control samples did not differ (Fig. 6G-I). This indicates that high concentration of small PS particles could trigger inflammation via innate immune system rather than via adaptive immune system. Along with confocal imaging result (Fig. 4A), we suggest that the immune cells are able to phagocytose PS particles and may have recognized them as pathogens. These results are consistent with previous studies which showed that PS particles less than 3 µm in diameter accelerate phagocytosis by increasing the production of cytokines, including IL-1 and IL-6. These cytokines are secreted by macrophages, which are related to innate immunity and inflammation^83,84^. IL-10 suppresses or regulates the inflammatory response of antigen presenting cells (APCs), such as dendritic cells and macrophages, and limits the adaptive response of CD4 + T cells. No increase in IL-10 secretion was observed under any of the experimental conditions (Fig. 6J–L). This indicated that the early stage of inflammation was triggered by the phagocytosis of PS particles by macrophage-like cells, and that the PS particles would not down-regulate the immune response. Although the effects of PS particles at lower concentrations and those of larger PS particles during the early stage of inflammation were less obvious, PS particles thus had the potential to cause toxicity by triggering inflammation in a size- and concentration-dependent manner.

#### Histamine profiles

The histamine assay was performed using PS particles with different sizes at a high concentration of 500 µg/mL, which induced IL-6 secretion (Fig. 6M). Unlike the results of a previous study on PP particles^49^, PS particles with different sizes did not generate differences in histamine release relative to the control. MHC-1 mast cells have a key function at the interface between innate and adaptive immunity, and they are primary effectors of immediate hypersensitivity. Mast cell-derived TNF-α has been reported to have a particularly important role in allergic inflammation. The secretion of IL-2 was not affected by treatment with PS particles in our study. However, this may indicate that PS particles cause acute inflammation without the involvement of histamines, and that they are more likely to activate innate immunity than adaptive immunity.

We conducted tests with HDFs and PBMCs to determine whether primary PS particles could cause inflammation and cytotoxic effects in humans without histamine mediation. PS particles with diameters of 0.46, 1, 3, 10, 40, and 100 µm and similar zeta potentials (1 ± 2 mV) were used for this experiment. The cytotoxicity results indicated that PS particles concentrations at <500 µg/mL did not reduce the viability of HDF cells and PBMCs. However, a high concentration (1,000 µg/mL) caused cytotoxicity in up to 50% of the HDF cells. PS particle size and surface charge were important factors in cytotoxicity. According to a recent study, NH_2_-labeled polystyrene nanoparticles were highly toxic to RAW 264.7 macrophages. Similar to our observations, the PS nanoparticles accumulated in the cytoplasm and induced calcium uptake in the mitochondria, which resulted in cell death^56^. Human macrophages could selectively phagocytose PS nanoparticles, particularly COOH-terminated PS particles. The THP-1 human monocytic cell line was more likely to endocytose the NH_2_-terminated PS nanoparticles. Another study showed that PS particles of various sizes accumulated in the livers, gills, and intestines of zebrafish and caused inflammation^85^.

## Conclusions

Although the migration of styrene monomers in foods and food contact materials (FCMs)^86^ is a concern, polystyrene products are useful for food packaging^87,88^ and are thought to be harmless. We confirmed that PS particles were not toxic to human cells at an experimental dosage of approximately 500 µg/mL. PS particles with diameters of 10–100 µm were not significantly cytotoxic. However, smaller PS particles with diameters of 460 nm and 1 µm affected RBCs. The small PS particles had larger surface areas than the larger PS particles. The adhesion of small PS particles to RBCs was enhanced by weak interactive forces, such as van der Waals forces, which led to hemolysis^64^. Most of the PS particles were located in the cytoplasm of HDF cells and PBMCs 24 hours after uptake. PS-FITC particles were observed in phagocytic PBMCs, such as neutrophils and macrophages, but not in lymphocytes. IL-2 secretion did not increase after treatment with PS particles under any of the experimental conditions. Therefore, PS particles seems not induce adaptive immunity secretion of TNF alpha increased after treatment with a high concentration of small PS particles, and IL-6 secretion increased after treatment with PS particles less than 3 µm in diameter at a concentration of 500 µg/mL. IL-6 is one of the main indicators of early-stage inflammation, so the intake of small PS particles may induce local inflammation in tissues and organs. We also measured IL-10 and histamine secreted by HMC-1 cells after treatment with ahigh concentration of PS particles. The PS particles did not cause an increase in histamine secretion. Thus, they were not likely to induce an allergic reaction orhistamine-mediated inflammation. The uptake of PS particles occurredmainly through end ocytosis and phagocytosis by phagocytic cells. This induced the release of pro-inflammatory cytokines that caused local inflammation rather than direct cytotoxicity. Smaller PS particles were generally not toxic to diverse human cells. However, direct contact with RBCs might cause hemolysis, and PS particles inhigher concentrations induced early-stage inflammation. The effects of secondary PS plastic particles and PS particles with different shapes should be studied in the future. To avoid the adverse effect of high concentration of PS nanoparticles, PS particles collected directly from the ocean and soil should also be monitored.

## Methods

The experimental protocol was reviewed and approved by the Institutional Animal Care and Use Committee at Chung-Ang University.

### Materials

#### All experiments were repeated at least three times with three samples each

Polystyrene particles labeled with fluorescein isothiocyanate (PS-FITC) with diameters of 460 nm were typically used as a label for detection, 1 µm, and 3 µm were purchased from SigmaAldrich (USA). PS particles with diameters of 10 µm, 40 µm, and 100 µm were purchased from Cospheric (USA). The PS particles were suspended in either PBS or cell culture medium prior to the experiment. Drabkin’s reagent, lipopolysaccharide (LPS), Triton X-100 (tx-100), and CCK-8 (Cell counting kits-8) cell counting kits were purchased from SigmaAldrich (USA). A human mast cell line1 (HMC-1) suspension was purchased from Merck Millipore (USA), and human dermal fibroblasts (HDFs) were obtained from Sigma (USA). Peripheral blood mononuclear cells (PBMCs) were purchased from CTL (USA). Due to human specimen control regulations, defibrinated sheep’s blood (Kisan Bio, Korea) was used instead. Human IL-2, IL-6, IL-10, and TNF-alpha (TNF-α) enzyme-linked immunosorbent assay (ELISA) kits were purchased from BioLegend (USA). Phosphate-buffered saline (PBS), Dulbecco’s phosphate-buffered saline (DPBS) without Ca^2+^ and Mg^2+^, and Roswell Park Memorial Institute (RPMI) 1640 medium containing 10% fetal bovine serum (FBS) were obtained from Gibco (Waltham, MA, USA).

#### Characterization of PS particles

PS particles were dispersed in PBS (pH 7.4), and their surface charges were determined *via* zeta potential analysis using a Zetasizer (Malvern Instruments, UK). SEM images of platinum-coated samples were captured using a field-emission scanning electron microscope (FE-SEM) purchased from Carl Zeiss (Oberkochen, Germany).

#### Cytotoxicity tests

Direct contact with PS particles has the potential to damage to skin. Human dermal fibroblasts (HDFs, Amsbio, USA) are the most ubiquitous cells in complex organisms. They are the predominant cells in stromal tissue, which plays an important role in the repair and healing of damaged organs by providing a protective barrier against PS particle absorption^89^. We used commercially available PBMC immune cellsto test the immunological response. The PBMCs were thawed, cultured for 16 h prior to use, and plated at 1 × 10^5^ cells per well in a 96-well plate. PS particles were added to the wells to final concentrations of 0, 1, 10, 100, 500, and 1,000 µg/mL. Cntl denoted no treatment, and tx-100 was added to the wells to serve as a positive control (n = 4). The PBMCs were cultured for four days in RPMI 1640, and HDFs were cultured in low-glucoseDulbecco’s Modified Eaglemedium (DMEM) with 10% fetal bovine serum. The cultures were prepared with 1% penicillin and streptomycin and incubated under 5% CO_2_ at 37 °C. Based on the hypothesis that people could be exposed to more than 20 mg of particles per year, we included high PS concentrations in the experimental design to observe how the particles affected cells. HDFs were seeded at a concentration of 5 × 10^4^ cells per well in a 96-well plate and treated with PS particles at different concentrations. Briefly, 10 µL of the CCK-8 reagent was added to the wells following addition of the PS particles. Absorbance was measured at 450 nm and compared to absorbance by the negative (N, no treatment) and positive (P) controls in 5% Triton X-100.

#### Confocal imaging

Prior to confocal imaging, the cells were fixed with 4% paraformaldehyde (PFA) for 1 h. They were then permeabilized with 1% Triton X-100 for 30 minutes, washed, and stained with 3 nM 4′,6-diamidino-2-phenylindole (DAPI) solution to make the nuclei distinguishable. Confocal images were obtained using a LSM 710 laser scanning microscope (Carl Zeiss, Oberkochen, Germany).

#### Hemolysis tests

*In-vivo* hemolysis tests were performed to identify severe acute toxic responses in RBCs induced by the PS particles. For this experiment, we assumed direct contact between the PS particles and the RBCs. We used sheep RBCs for the study, because their structures and functions were similar to those of human RBCs. Sheep’s blood (20 mL) was mixed with 20 mL PBS, then centrifuged at 500 *g* for 30 minutes. All of the supernatant was removed *via* aspiration. The purified RBCs were mixed with PBS in a 1:2 (v/v) ratio, and 1 mL aliquots of the RBC mixture were treated with PS particles in final concentrations of 0, 1, 10, 100, 500, and 1,000 µg/mL. The mixtures were agitated on a rotary mixer for 12 h. After the reaction, the supernatants were collected and mixed with Drabkin’s reagent in a 1:1 (v/v) ratio. The mixtures were held at room temperature for 20 minutes, and the degree of hemolysis was analyzed by measuring UV absorbance at 540 nm.

#### Cytokine profiling

We focused on the induction of pro-inflammatory cytokine expression in HMC-1 and PBMC samples prepared in 200 µL aliquots of the culture medium. The supernatants were collected four hours after treating the cells with PS particles. Interleukin 6 (IL-6) secretion in the early stages of PS treatment could indicate a relationship between IL-6 and the inflammatory response. HMC-1 cells were cultured to 80% confluency in T-25 flasks. PS particles with sizes ranging from 460 nm to 100 µm were added, and the ELISA assay was performed to evaluate total histamine expression. The PBMCs provided unique information about the expression of constituent cytokines that could indicate an immune response against PS particles *in vivo*. The PBMCs were thawed and cultured for 16 h prior to the experiment. We then plated 5 × 10^5^ cells per well in a 24-well plate, and PS particles were added to final concentrations of 0, 10, 100, and 500 µg/mL. The plate was incubated for four days, and 2.5 µg/mL LPS was used as the positive control. The ELISA assay was performed according to manufacturer’s protocol.

#### Histamine profiling

Histamines, including the catecholamines dopamine and epinephrine and spermine, a polyamine, are produced as part of the local immune response underlying histamine-mediated inflammation. Histidine is converted to histamine by histidine decarboxylase, which is stored in granules in mast cells and basophils^90,91^. Histamine is released from the granules and is known to contribute to allergic reactions, asthma, eczema, and coughing. It plays an important role as a chemical mediator to accelerate mucus production in glands, bronchial smooth muscle contraction, hypertrophy, and the enlargement of peripheral blood vessels^92,93^, histamine release is induced by reactions between the antibodies on mast cells and antigens. Histamine alsohas a critical role in the Ig E-mediated allergic response. Therefore, histamine secretion *in vitro* was evaluated to determine whether or not the PS particles were allergens^93^. The HMC-1 cells were thawed, seeded at 5 × 10^5^ cells/mL in a 96-well plate, and incubated for 24 h. They were then treated with PS particles with different sizes and concentrations for 48 h. After treatment, 200 µL of each supernatant was collected and analyzed using a histamine ELISA kit according to the manufacturer’s protocol.

### Statistical analysis

GraphPad Prism software^94^ was used for statistical analysis and graphical representation of the data. ImageJ^95^ was used to determine the particle count from optical microscope images and to estimate the fluorescence intensity in the experiments. Student’s *t*-tests and ANOVA were performed to analyze the data. Non-significant values are indicated by NS in the results section. *P*-values of less than 0.05 and 0.001 are indicated by * and **, respectively.

### Ethics Approval and Consent To Participate

No tests, measurements, or experiments were performed on humans in this work.

## Data Availability

The datasets supporting our conclusions arepresented in the article.
